# Epidemiologic Features of Type 1 Diabetic Patients between 0 and 18 Years of Age in İstanbul City

**DOI:** 10.4274/jcrpe.1694

**Published:** 2015-03-05

**Authors:** Fikri Demir, Hülya Günöz, Nurçin Saka, Feyza Darendeliler, Rüveyde Bundak, Firdevs Baş, Olcay Neyzi

**Affiliations:** 1 Dicle University Faculty of Medicine, Department of Pediatrics, Diyarbakır, Turkey; 2 İstanbul University Faculty of Medicine, Department of Pediatric Endocrinology, İstanbul, Turkey

**Keywords:** Type 1 diabetes in children, epidemiology of type 1 diabetes mellitus, diabetic ketoacidosis, seasons, autoantibodies, Turkey

## Abstract

**Objective:**

To evaluate the epidemiologic, clinical and laboratory characteristics of a group of children with type 1 diabetes mellitus (T1DM) living in a Turkish city.

**Methods:**

The records of 395 (boys/girls: 199/196) children with newly diagnosed T1DM hospitalized in the years 1985-2004 were evaluated retrospectively. The data were assessed by gender and age subgroups (≤5, 6-10 and ≥11 years).

**Results:**

Mean age of children at diagnosis was 8.1±4.1 years. At T1DM onset, the number of children ≤5, between 6-10 and ≥11 years old was 110 (27.9%), 147 (37.2%) and 138 (34.9%), respectively. The patients were mostly diagnosed at ages 6-8 years (24.1%), followed by cases aged 3-5 years (22.0%). Polyuria and polydipsia were the most common symptoms (94.7%). Mean duration of symptoms was 21.5±18.6 days. Although the patients mostly presented in autumn (30.7%), no season-related significant differences were found. The frequency of ketoacidosis was relatively high (48.5%). When compared to boys, the girls experienced higher rates of ketoacidosis (55.1% vs. 41.7%, p=0.042); had a higher frequency of anti-thyroid peroxidase antibodies (11.7% vs. 4.2%, p=0.049) and higher insulin requirement (0.89±0.41 vs. 0.77±0.36 IU/kg, p=0.005). Cases with a family history of T1DM were more likely to have anti-endomysial antibodies (42.9% vs. 8.1%, p=0.027) and higher initial blood glucose levels (510.5±145.0 vs. 436.1±156.5 mg/dL, p=0.005).

**Conclusion:**

The findings possibly indicate a decreasing age of T1DM onset. The high frequency of ketoacidosis at presentation is noteworthy. Girls had higher rates of ketoacidosis, higher frequency of anti-thyroid antibodies and higher insulin requirements as compared to boys. Patients with a family history of T1DM had higher initial glucose levels and higher frequency of anti-endomysial antibodies.

## INTRODUCTION

Type 1 diabetes mellitus (T1DM) is an autoimmune disease characterized by immune-mediated damage to pancreatic islet β-cells and resultant progressive insulin deficiency (1). The clinical consequences of insulin inadequacy vary from polyuria and polydipsia to diabetic ketoacidosis (DKA), altered consciousness and if not treated, to death (1,2). Prolonged hyperglycemia results in macro- and/or microangiopathic involvement and long-term complications associated with significant morbidity such as retinopathy, nephropathy, neuropathy and coronary atherosclerosis and consequently with mortality (3).

The epidemiologic features of T1DM have been changing. The incidence of the disease has increased and the age of onset has decreased in recent years, albeit the reasons are yet unknown. Early diagnosis is important in order to prevent the acute and chronic complications of T1DM (4,5,6). Previous epidemiologic studies concerning T1DM, especially of large cohorts, have been useful for recognition and comparison of changing characteristics and establishment of novel management strategies (1,2,7). However, in Turkey, studies on the epidemiologic features of T1DM are limited (8,9,10) and only one of these studies was conducted on a large cohort (8).

In this paper, aiming to provide data relating to the changing characteristics of T1DM, the clinical, epidemiologic and some laboratory characteristics of a group of T1DM patients from a large metropolitan area are presented.

## METHODS

Newly-diagnosed 0 to 18 years old T1DM patients hospitalized in the Pediatric Endocrinology Clinic of İstanbul University İstanbul Faculty of Medicine between the years 1985 and 2004 were included in the study. The records of these patients were retrospectively reviewed. Patients whose records were inadequate were excluded. The study was approved by the Institutional Ethics Committee.

Demographic and medical characteristics obtained from the records included date of birth, age at T1DM diagnosis, complaints, duration of symptoms, season of presentation, season of birth, physical findings, height and weight measurements and laboratory findings including urinary ketone content, results of blood biochemistry which included blood glucose, blood pH, hemoglobin A1c (HbA1c) levels, islet cell antibodies (ICA), insulin autoantibodies (IAA), anti-glutamic acid decarboxylase antibodies (GADA), anti-thyroid peroxidase and anti-thyroglobulin antibodies, serologic findings of celiac disease including anti-gliadin and anti-endomisium antibodies, the presence of human leukocyte antigen (HLA)-DR3 and -DR4. Daily insulin dose and administration protocol applied were also recorded. These data were assessed in the total study population and also by gender and age subgroups (≤5, 6-10 and ≥11 years).

Most of the patients had been hospitalized for 7-10 days in order to adjust their daily insulin doses and to educate both patients and their families about management of T1DM. Only zinc-crystallized and neutral protamine hagedorn (NPH) insulins were available during the study period. Insulin doses were administered as 2, 3 or 4 daily injections.

Body mass index (BMI) was calculated as weight (kg)/[height (m)]2. Standard deviation scores for BMI (BMI SDS) were calculated using previously reported data on Turkish children (11). Since the current study did not aim to perform an anthropometric evaluation of the patients and since the age range of the cases was too large, only BMI and BMI SDS values were used in the assessment.

The patients with a fasting blood glucose level ≥126 mg/dL or a glucose level ≥200 mg/dL regardless of meals and without ketosis and acidosis were included in the hyperglycemia group. Those with hyperglycemia and ketosis but without acidosis were included in the ketosis group and the children with hyperglycemia, ketosis and acidosis (pH <7.25) constituted the ketoacidosis group. A pH value of less than 7.10 was evaluated as severe acidosis. HbA1c levels of ≤6% were considered as normal. A temporary reduction in insulin requirement is usually observed following T1DM diagnosis. In this period, patients with an insulin requirement below 0.2 units/kg/day were accepted to be in complete remission, while an insulin requirement between 0.2-0.5 units/kg/day was considered as partial remission.

The data were processed and analyzed with SPSS 15.0 statistical package software (SPSS Inc., Chicago, Illinois, USA). Distribution pattern of all data was evaluated by Kolmogorov-Smirnov test. Qualitative variables were shown as numbers and percentages and quantitative variables were demonstrated as means ± SD. The chi-square test and Fisher’s exact test were used to compare the qualitative data. The differences between quantitative groups with normal distribution were evaluated with student’s t-test or the analysis of variance (in case of significant differences further subgroup analyses were performed with Tukey’s test). The differences between quantitative groups with abnormal distribution were evaluated with either Mann-Whitney U test or Kruskal-Wallis test. A p-value of <0.05 was considered as statistically significant.

## RESULTS

**Figure 1:**
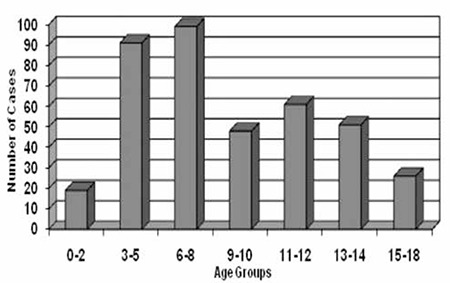
Distribution of the cases by age

The characteristics of the study population by age subgroups and gender are shown in Tables 1 and 2. The total number of the sample was 395 (199 boys and 196 girls) and their mean age at diagnosis was 8.1±4.1 years. At the time of diagnosis, 110 of the children (27.9%) were younger than age 5 years, 147 (37.2%) were between ages 6 and 10 years and the remaining 138 cases (34.9%) were between ages 11 and 18 years. The first peak in the number of T1DM cases in terms of age at diagnosis was observed in the age group between 6 and 8 years [n=95 (24.1%)] and it was followed by a second smaller peak in the age group between 11 and 12 years [n=54 (13.7%)]. The number of cases between 3 and 5 years was quite close to the ones in the age group between 6 and 8 years and higher than the remainder age groups [n=87 (22.0%)] (Figure 1). BMI SDS did not differ by age subgroups or gender. Twelve of 274 children (4.4%) with relevant data were obese (BMI SDS ≥2). Mean duration of symptoms in the total study population was 21.5±18.6 days. Although a longer duration of symptoms was reported in older cases and in girls, no significant differences were determined. Polyuria and polydipsia were the most common complaints accounting for 94.7% of 378 cases with adequate information in their medical records. Tachypnea was observed significantly more commonly in cases younger than five years (p=0.001). Other symptoms did not differ by age or gender. Family history revealed T1DM in 8.4%, T2DM in 41.1% and both types in 1.8% of the patients. No difference was found concerning family history of DM between age subgroups and gender. However, the cases with a family history of T1DM were more likely to be positive for anti-endomysial antibodies (42.9% vs. 8.1%, p=0.027) and to have higher initial blood glucose levels (510.5±145.0 vs. 436.1±156.5, p=0.005).

As shown in Tables 1 and 2, mean HbA1c level of the total study population was 10.5±2.6%. HbA1c levels were normal in 4.8%, higher than 10% in 55%, between 8-10% in 26.9% and between 6-8% in the remainder of the cases. HbA1c levels did not differ by age subgroups or gender. Almost half of the cases (48.5%) presented with DKA, 35.4% with ketosis and 16.1% with hyperglycemia. While there were no significant differences between the age subgroups, gender-related evaluation revealed a higher frequency of DKA in girls (55.1% vs. 41.7%, p=0.042). When the study population was evaluated according to year of admission, the cases who had presented within the past five years were found to have a lower frequency of ketoacidosis than previously admitted patients at a borderline level of significance (42.6% vs. 55%, p=0.06). Frequency of severe DKA was also found to have decreased from 16.2% to 15.6% in recent years.

The patients presented most frequently in autumn months (30.7%), followed by winter months (28.3%) (Table 3). Interestingly, summer was the season in which most of the cases younger than five years presented (30.4%) and the difference was significant (p=0.049). As to season of birth, most of our cases were born in summer months (30.7%).

Data on HLA subgroups were available in 53 cases. Frequency of HLA-DR3 and -DR4 were 58.5% and 52.8%, respectively in these patients. Most of the cases (88.7%) were positive for at least one of the HLA groups and 22.6% were carrying both HLA subgroups. Islet cell antibody was present in 44.4% and insulin autoantibody was found in 42.6% of 244 children with adequate related data. We did not observe any difference in autoantibody or HLA positivity among age or gender subgroups (Tables 1 and 2). The frequency of any anti-thyroid antibody positivity was 11.4% (23/202) in T1DM cases with related data. Anti-thyroid antibodies were observed more frequently in girls. However, significant differences were determined only for presence of thyroid peroxidase antibody and coexistence of both antibodies (7.6% vs. 1%, p=0.036). Although no difference of statistical significance was found, the frequencies of anti-thyroid peroxidase and anti-thyroglobulin antibodies were higher in younger (≤5 years) children. Anti-gliadin and anti-endomisium antibodies were detected in 14.6% and 10.8% of cases, respectively. Anti-gliadin and anti-endomisium antibody positivity did not differ by gender or age.

Insulin requirements, administration protocol and state of the patient at hospital discharge are shown in Tables 1 and 2. Mean daily insulin dose was 0.82±0.39 IU/kg. Boys needed a significantly lower insulin dose than girls (0.77±0.36 vs. 0.89±0.41 IU/kg, p=0.005). Partial and complete remission rates were 62 (18.8%) and 10 (3.0%), respectively, in the 330 cases with the relevant information in their files. Remission was observed less frequently in children older than 10 years (p=0.025).

## DISCUSSION

T1DM is one of the most common chronic endocrinologic diseases of childhood with a changing epidemiology in terms of decreased age of onset and increased frequency for yet unknown reasons. A good glycemic control is crucial to avoid or delay chronic complications of T1DM (3,4,5). In this study, the clinical presentation and baseline characteristics of a relatively large cohort of children with T1DM were evaluated and it was observed that the number of preschooler cases (3-5 years old) was quite close to that of the age subgroup with highest number (6-8 years old). We believe that this patient population may be quite representative of Turkey due to its heterogeneous demographic structure related to excessive emigration to a metropolis (İstanbul city) from all around the country.

Age of onset of T1DM showed a bimodal distribution. The first peak was attributed to increased frequency of infections in the early school years and the second one was thought to result from pubertal stress, related to insulin antagonism of growth hormone and gonadal hormones (12,13). Similarly, evaluation of our patients according to age subgroups revealed that the number of cases increased after the first two years of life and peaked in the age group between 6 and 8 years. This was followed by a second smaller peak in the 11 to 12 years age group. Interestingly, the number of cases between ages 3 and 5 years was found to be quite close to that of the age group of 6 to 8 years. This finding may be related to a pronounced increase in the incidence rates of T1DM in younger children, a finding previously reported by others (4,5,6,14). Besides the possibility that smaller children are referred to a university center more frequently, this finding may also be related to increased rate of kindergarten enrollment and to associated higher prevalence of triggering infections. However, in contrast to our findings, another study from Turkey found that most of their cases presented between ages 12 and 14 years and these authors did not report a noteworthy increase in the frequency of cases younger than 5 years old (8). That study was conducted between the years 1969 and 1991, approximately 15 years preceding our study and this difference in age of onset may be reflecting a tendency to an ongoing decrease in age of T1DM onset.

It was stated that T1DM cases presented generally in cold seasons because islet cells were damaged initially by viral infections and an autoimmune response was responsible for ongoing injury (12,15,16). We had observed a similar seasonality pattern in our population, in line with a previous study (10). However, contradictory results were also reported - some authors found higher rates of presentation in winter but not in autumn (8), while others reported spring or summer as the seasons in which most of the cases were diagnosed (9,17).

We found a family history of T1DM and T2DM in 10.2 and 42.9% of the patients, respectively. Frequency of T1DM family history was similar to the findings of Kandemir et al (10.3%) (8). Our results regarding the frequency of a family history in T2DM was very similar to those reported by Demirbilek et al (39%) (9), but the frequency of a family history in T1DM by these authors was higher (26.8%). Although cases with positive family history were reported to be diagnosed at an earlier stage, generally without ketoacidosis and with a shorter duration of symptoms (2,18), our results did not agree with these findings. Pawlowicz et al (19) also reported that a positive family history had no such impact. The causes of higher initial glucose levels and increased frequency of endomysial antibody positivity in the cases with family history of T1DM remain to be explained and warrant further evaluation.

Metabolic decompensation in T1DM results in ketoacidosis and cerebral edema which are the leading causes of diabetes-related death in childhood. The frequency of DKA at presentation was reported as 26% to 67% previously. Ketoacidosis may also develop during follow-up because of inadequate compliance with insulin treatment or increased insulin requirement during illnesses. DKA has been reported to be more frequent in underdeveloped populations and also in countries with low T1DM incidence (7,12,17,20,21). Interestingly, a relatively high rate of DKA was observed in our population (48.5%), but it was not as high as in some centers from Romania (67%) and Poland (54%) (7). Severe DKA was seen in 15.9% of our cases. The relatively high DKA frequency in this study population may be related to lack of sufficient awareness in a noteworthy number of people living in underdeveloped areas and also to the relatively lower prevalence of T1DM in our country (0.67/1000 cases) (22). In a study from an underdeveloped region of Turkey with a population of low socio-economic level, even higher rates for DKA and severe DKA have been reported (65.9%/41.5%) (9). Furthermore, the finding that patients who presented within the past five years had a relatively lower frequency of DKA (42.6% vs. 55%) may indicate an improvement associated with the efforts of the Turkish Ministry of Health and of non-governmental organizations in raising the awareness of the community. The girls were stated to experience DKA more frequently, possibly due to some sex-related social or biological differences (23). We also found significantly higher rates of DKA in girls (55.1% vs. 41.7%, p=0.042). However, another study from our region did not report such a distinction (9). Albeit not significant, longer duration of symptoms determined in girls may be another cause of higher frequency of metabolic decompensation. Although relatively lower rates of DKA were reported in cases with a family history of T1DM (18), our findings did not reveal a significant impact of family history on incidence of DKA (43.5% vs. 49.3%). The findings reported by Demirbilek et al (9) were similar to ours.

T1DM is an autoimmune condition commonly accompanied by other autoimmune diseases such as thyroiditis and celiac disease, possibly because of some common pathogenetic mechanisms including certain gene expressions (12,24). These autoimmune abnormalities are observed more frequently in females with T1DM (12,25). Although no gender-related difference was determined in anti-gliadin and anti-endomisium antibody positivity in our series, anti-thyroid antibodies were more common among our girl patients.

The incidence of T1DM in younger children has been increasing in our country in parallel with the global trend (5,22). The younger diabetic cases were reported to have a shorter duration of symptoms (12,18). Indeed, our younger patients had a relatively shorter duration of symptoms, but the difference was not statistically significant. Some studies reported a different seasonality pattern in younger patients and stated that they were mostly diagnosed in summer (5,16). We also found a significant tendency of younger patients to be diagnosed in summer months. Although the reason for this seasonal difference is not completely understood, it may be associated with various environmental triggers including infections encountered more frequently in the younger age groups due especially to kindergarten enrollment. A more abrupt and severe clinical course in children with DKA younger than 5 years was also reported (2). However, a multicenter study that found a higher frequency for DKA in areas with low T1DM incidence did not report any difference in clinical course in younger children (7). In the current study, we found a relatively higher ratio of DKA in younger children, but the difference was not significant. Increased respiratory rate was observed more commonly in cases younger than 5 years. Possibly due to the physiologically lower lung capacity of younger children, the respiratory rate increases at an earlier stage and this may serve to draw attention to the severity of the clinical condition. In contrast to the authors stating no age-related difference (26,27), a number of investigators suggested that T1DM children with increased tendency to autoimmune thyroiditis presented at relatively younger ages (28). Interestingly, although this finding lacked statistical significance, the frequencies of autoantibodies associated with thyroiditis were also higher in our younger patients. Other features of T1DM, such as family history, frequencies of ICA, IAA, GADA autoantibodies, HLA subgroups and other accompanying autoimmune conditions did not differ significantly in our younger diabetic cases. Accelerated growth was accused of predisposing to T1DM and some studies reported relatively higher BMI SDS in T1DM cases diagnosed at younger ages (accelerator hypothesis) (29,30). However, we did not find higher BMI SDS values in our younger population, nor did Porter et al (31).

Various parameters including age, gender, BMI and mode of insulin application have been reported to affect the daily insulin requirement (32,33). Some studies have suggested that insulin requirement increases in adolescents due to the antagonism of growth hormone and gonadal hormones (12,13). We also found relatively higher insulin requirements in older children, but this finding had no statistical significance. In line with some previous reports (32,34), our male cases required less insulin than girls. This may be associated with sex-related differences in adipocytokine release, insulin sensitivity and hepatic clearance of insulin (35).

In conclusion, the findings of the study possibly indicate a decreasing age of T1DM onset. The girls were found to have higher rates of ketoacidosis, higher frequency of anti-thyroid antibodies and higher insulin requirements as compared to the boys. Patients with a family history of T1DM had higher initial blood glucose levels and a higher frequency of anti-endomysial antibodies. Despite increasing communal awareness, the prevalence of DKA in Turkish children with T1DM is still high. This finding points to a need for measures for the timely diagnosis of T1DM to prevent life-threatening events.

## Figures and Tables

**Table 1:**
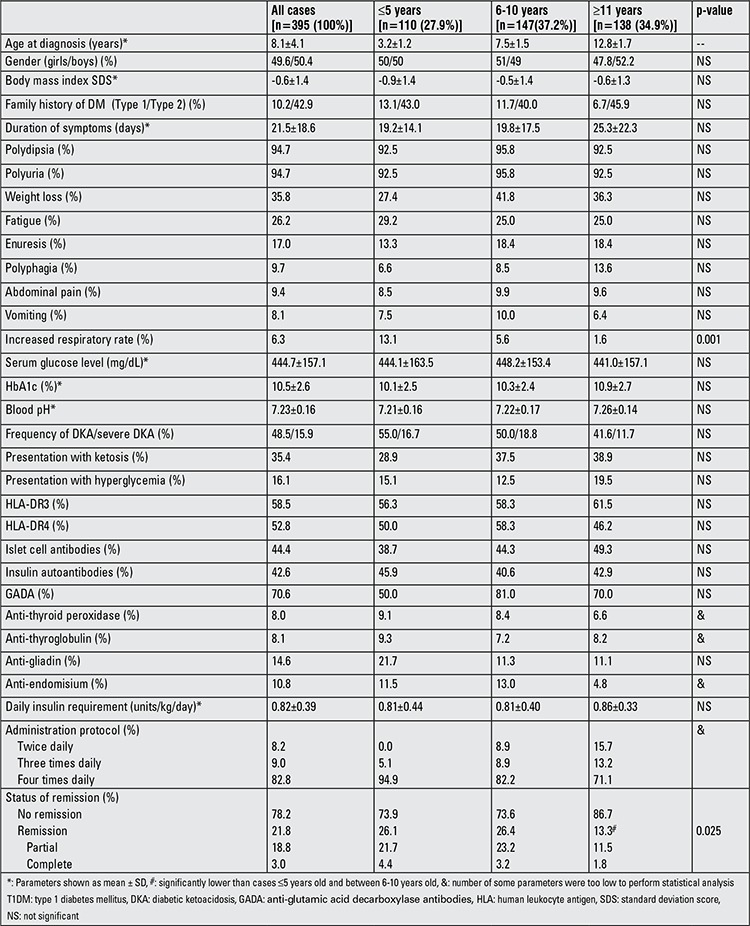
The characteristics of T1DM patients in the age subgroups

**Table 2:**
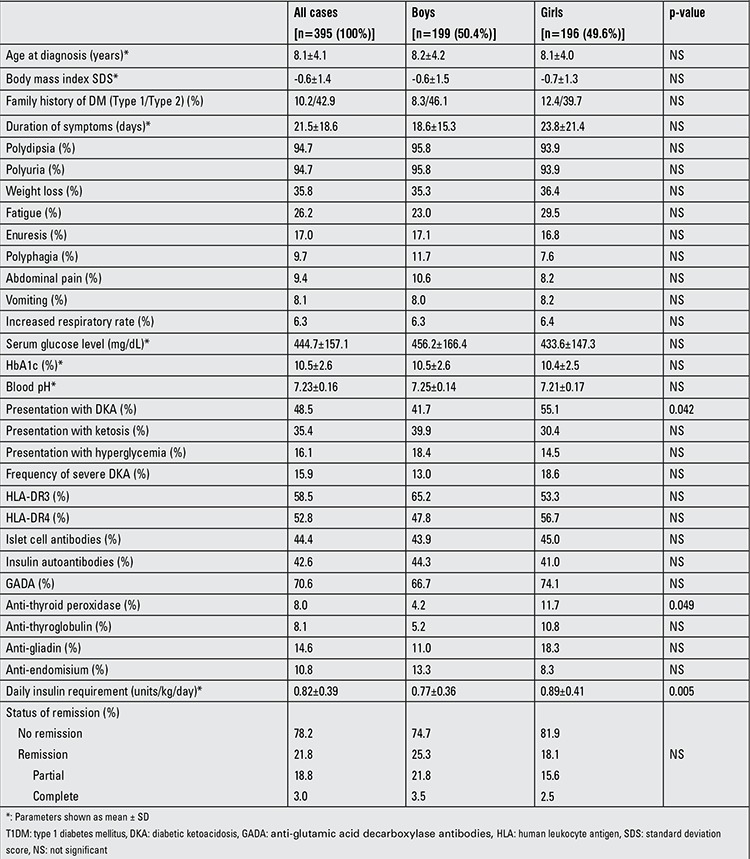
The characteristics of T1DM patients by gender

**Table 3:**
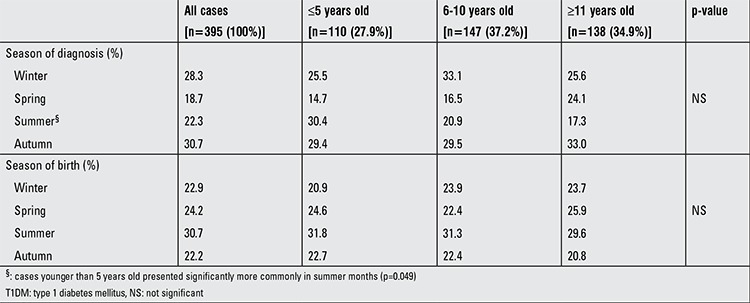
Characteristics of T1DM patients by season of the year at diagnosis and at birth
